# Sexual and reproductive health communication between parents and high school adolescents in Vientiane Prefecture, Lao PDR

**DOI:** 10.1080/16549716.2020.1785145

**Published:** 2020-08-03

**Authors:** Visany Vongsavanh, Vu Thi Hoang Lan, Vanphanom Sychareun

**Affiliations:** a Faculty of Public Health, University of Health Sciences, Vientiane Capital, Lao PDR; b Department of Postgraduate Education, Hanoi University of Public Health, Hanoi, Vietnam; c Faculty of Fundamental Sciences, Hanoi University of Public Health, Hanoi, Vietnam

**Keywords:** LEARN: Sexual Reproductive Health, ANC and Nutrition, Sexual reproductive health, communication, adolescents, Lao PDR

## Abstract

**Background:**

Adolescent health has become a priority on the global health agenda. Parent-adolescent communication regarding sexual and reproductive health (SRH) issues can help to reduce adolescent risk-taking sexual behaviours.

**Objective:**

This study was to describe the situation of SRH communication, and to determine the factors associated with SRH communication between high school students and their parents in Vientiane Capital prefecture, Lao PDR.

**Methods:**

A multistage sampling technique was applied. A self-administered questionnaire was implemented among a sample of 384 high school students aged 14–17 in Vientiane. SRH communication in this study was recorded as the frequency with which adolescents discussed with their parents at least four topics on SRH issues during a six-month period prior to the interview. Data were entered and analysed using Epi Data software version 6.0 and STATA software version 14.2.

**Results:**

Slightly more than one-fifth of the students (21.3%) communicated with parents on SRH issues. The multivariate logistic regression model showed that being a male adolescent (AOR = 2.1; 95% CI 1.2 to 3.5), urban school locations (AOR = 0.2; 95% CI 0.1 to 0.5), a mature father (AOR = 1.7; 95% CI 1.0 to 2.9), positive attitudes towards general communication with parents (AOR = 2.2; 95% CI 1.1 to 4.2) and accessibility to multiple SRH information sources (AOR = 5.2; 95% CI 2.4 to 11.4) were significantly associated with adolescent-parent communication on SRH issues.

**Conclusion:**

This study showed that student-parent communication on SRH issues was low, so policymakers should develop programs to improve SRH communication skills in all schools and encourage open discussion among family members, especially with respect to the participation of adolescent girls. The positive attitudes of students and multiple sources of SRH information were also important factors in improving SRH communication.

## Background

Adolescent health has become a priority on the global agenda with an added focus on addressing adolescent health in low- and middle-income countries if the Sustainable Development Goals (SDGs) are to be reached [1]. Adolescence is defined as the period of life between 10 and 19 years of age [2]. It represents a period of life characterised by significant physical, cognitive, emotional and social changes [3] as children transition to adulthood [4]. Traditionally in Lao PDR, as in many Asian cultures, sexual behaviour before marriage has been considered highly inappropriate, with parents emphasising abstinence [5], but nevertheless, many adolescents are sexually active while unmarried or out-of-union. Lao PDR, a lower-middle income country in South-East Asia, has one of the highest adolescent pregnancy rates among countries in the region; at 83 per 1,000 adolescent girls aged 15–19 being pregnant [6]. Early childbearing is common with about 3.6% of women giving birth by age 15 [7], and more than 1 in 10 girls aged 15–19 have begun childbearing [8]. In addition, abortion is common in Vientiane, with over 20% of sexually active young women reported to have had an abortion, often in unsafe conditions [9]. Additionally, the new cases of HIV among youths aged 15–24 years in Vientiane were 16.7%, 18.8% and 15.9% in each respective year for 2010, 2011 and 2012 [10].

Parent-adolescent sexual communication can positively affect safer sexual behaviour among adolescents, including utilisation of modern contraceptive methods. Through discussion of adolescent sexual activity, parents can influence on adolescents’ sexual attitudes, values and beliefs that help reduce sexually risky behaviour [11,12]. For parent-adolescent sexual communication to be effective however, parents need to be able to communicate openly with and give accurate and correct advice about SRH to their children as they become more sexually aware and active [11,12]. Additionally, adolescents need to feel comfortable when discussing reproductive health with their parents including talking about the physical changes at puberty, the menstrual cycle, wet dreams, birth control pills and condom use [13,14]. When adolescents and partners feel uncomfortable or not empowered to engage in open discussion about sexual health, they may become involved in risky sexual behaviour at an early age, which may result in unwanted pregnancies, abortion (sometimes in unsafe conditions) as well as exposure to sexually transmitted infections (STI) including HIV/AIDS [15,16]. Despite the potential benefits of parent-adolescent sexual communication [17], studies have found many adolescents have not discussed sexual topics with a parent. Studies have shown that only 20%, 36.9% and 30.6% of adolescents respectively in Lesotho, Ethiopia and China had discussed SRH issues with their parents [18–20]. The barriers to discussing these topics with parents for adolescents included parents’ lack of knowledge, negative attitudes and socio-cultural taboos [21].

SRH communication may protect adolescent children from SRH issues, but there is little research on what helps adolescents initiate the conversation about SRH matters with their parents [22]. The significance of this study is that it will help to design appropriate interventions to improve open discussions with parents to prevent different adverse SRH issues for adolescents. Therefore, this research aimed to study adolescent-parent communication on SRH issues and determine factors associated with general and SRH communication between adolescents and their parents in Vientiane.

## Methods

### Study design and setting

This study employed a cross-sectional design with a multi-stage sampling technique. First, it selected one school from 14 public high schools in four urban districts (Chanthabouly, Sikhottabong, Sisattanak and Xaysettha) and one school from five public high schools in one rural district (Sangthong) by random sampling due to limited time and budget, with one urban-based High School and one rural-based High School selected. Next, proportional sampling was used to select the number of classes in each grade. Finally, all students in the selected classes were invited to participate in the study.

### Participants

The population size was 950 adolescent students in grades 9 to 12 at one High School in urban and the one High School in rural area. The sample size was 384 which was determined using the single population proportion formula when considering the following assumptions p = 0.5 (it was hypothesised that the percentage frequency of outcome in the population was 50% for the estimated proportion of students communicating on SRH issues with parents due to unknown the proportion of the proportion of SRH communication between adolescents and parents in Lao PDR); d = 0.05 (a confidence level of 95% and a margin of error of 5%); Z (∝/2) = 1.96 (a significance level of 5% (α = 0.05)). To this was added a 10% non-response rate and design effect of 1.3.

### Measurements

The independent variables included four sections: (1) socio-demographic factors (age, grade, sex, school location, parent’s marital status, living arrangement, fathers and mothers age, education and occupation), (2) the attitudes of students, (3) influence person (grandparent, father, mother, sister, brother, teacher, peer, neighbour, health staff) and (4) SRH information sources (school, health facility, youth centre, internet, TV, radio, newspaper, magazine).

The questionnaire about attitudes towards general and SRH communication was validated according to the Parent–Adolescent Communication Scale [16,23] and translated into Lao. Each question was answered on a four-point Likert Scale of attitude statements ranging from 1) strongly disagree, 2) disagree, 3) agree and 4) strongly agree. However, there were four negative questions which were answered with 4) strongly disagree, 3) disagree, 2) agree and 1) strongly agree.

The attitudes towards general communication with parents constituted 15 questions. The scale of reliability coefficient for the attitudes towards general communication with parents was 0.816. A summated composite score was calculated with a minimum score of 15 and a maximum of 60. The actual scores were classified base on Bloom’s criteria [24] and modified into two groups as follows: scores of 15–27 (<60%) were regarded as the respondents indicating a negative attitude and scores of 28–60 (>60%) were regarded as their displaying a positive attitude. The attitudes towards SRH communication with parents included 22 questions. The scale of reliability coefficient for the attitudes towards sexual and reproductive health communication with parents was 0.808. A summated composite score had a minimum score of 22 and a maximum score of 88. Actual scores were classified base on Bloom’s criteria [24] and modified into two groups as follows: scores of 22–39 (<60%) were regarded as the respondents indicating a negative attitude and scores of 40–88 (>60%) were regarded as their showing a positive attitude.

The dependent variable for the questionnaire was measured by the frequency communication between adolescents and parents on SRH issues by incorporating the eight questions related to SRH communication with the father and eight questions related to SRH communication with the mother. This was assessed using a Likert Scale of eight items/topics with responses ranging from 0) never = never per six months, 1) rarely = 1 time per six months, 2) sometimes = 2 times per six months to 3) often more than 2 times per six months which was derived from the weighted Topics Measure of Family Sexual Communication Scale [25,26]. The items included physical changes at puberty, menstrual cycles/wet dreams, premarital sex, multiple sex partners, unwanted pregnancies, birth control pills, condom use, STDs/HIV/AIDS. Responses to each of these eight items were classified into two groups: No = (never and rarely) = 0 and Yes = (sometimes and often) = 1. Then, a summated composite score was produced through totalling all items, and the Cronbach’s alpha for the internal consistency was 0.903. For the purpose of this analysis, parent-adolescent SRH communication was regarded as existent if the adolescents and their parents discussed at least four of eight SRH topics sometimes or frequently [27].

### Data collection

The data collection team included eight people who were trained on the objectives of the study, the content of the questionnaire and how to conduct the study in a high school setting. After that, the team visited each selected school and identified eligible students. Two research assistants were allocated per class and explained the purpose of the research to the students and the content of each section of the questionnaire. The students completed the self-administered questionnaire, and the research assistants checked the completeness of all answers. If there were missing answers, the research assistants returned the questionnaire to complete the missing questions. After checking all questionnaires, the students were allowed to leave the classrooms.

### Statistical analysis

The data were entered and cleaned using Epi Data software version 6.0. The data were analysed using STATA version 14.2. Descriptive statistics was used to describe the numbers and percentages of the dependent and independent variables. Then, the bivariate analysis was performed between the independent variables and the dependent variable. The variables in the bivariate analysis found to be significant at p < 0.05 were entered into the multivariable logistic regression model. In the multivariate analysis, standard data analysis techniques were applied. Variables having p < 0.05 in the multivariate analysis were taken as significant predictors. Crude and adjusted odds ratios with their 95% confidence intervals were calculated and presented in texts and tables.

## Results

In total, 384 students aged 14–17 in grades 9 to 12 were enrolled into the study. About 63% of the respondents were from an urban district and slightly higher than half were females, in addition the mean age of respondents was 15.7. The reported mean age of their fathers was 47.2 and 40.6% of their fathers had graduated from the tertiary education level. Their fathers were mostly employed as government staff or private businesses. In addition, the mean age of their mothers was 42.6 and 41.9% of mothers had graduated from high school or vocational college. About 27.1% of mothers were housewives (Table 1).

**Table 1.** Socio-demographic characteristics of students and their parents in Vientiane Capital.

Variable	Total (n = 384)	
	Number	Percentage (%)
**Age**		
14–15 years old	158	41.1
16–17 years old	226	58.9
**Sex**		
Female	230	59.9
Male	154	40.1
**School location**		
Rural	142	37.0
Urban	242	63.0
**Marital status of parents**		
Together	327	85.2
Separated/Divorced	57	14.8
**Living arrangement**		
Both parents	316	82.3
Mother only or father only	47	12.2
Relative or friend	21	5.5
**Age of father**		
47 years or below	206	53.6
Above 47 years old	178	46.4
**Father’s education**		
Primary	87	22.7
High school/Vocational	141	36.7
Tertiary	156	40.6
**Father’s occupation**		
Unemployed	18	4.7
Employee (private/govt)	182	47.4
Other jobs	184	47.9
**Age of mother**		
42 years or below	176	45.8
Above 42 years old	208	54.2
**Mother’s education**		
Primary	133	34.6
High school/Vocational	161	41.9
Tertiary	90	23.5
**Mother’s Occupation**		
Housewife	104	27.1
Employee (private/govt)	103	26.8
Other jobs	177	46.1

### Frequency of adolescent–parent communication on SRH issues

About 21.3% of students in this study had discussed at least four topics in eight topics for SRH issues with their parents during the six months prior to the survey. Of these students, male adolescents discussed the topics more frequently with their parents than female adolescents (29.2% versus 16.1%). Moreover, male participants also discussed SRH issues with their father and their mothers more often than female participants. These results highlight that boys talk more than girls to both their parents about SRH issues. However, more adolescent boys and girls talk with their mothers than with their fathers (Table 2).

**Table 2.** Communication on SRH issues between adolescents and parents in Vientiane Capital.

SRH communication	Male (n = 154)	Female (n = 230)	Total (n = 384)
	Number (%)	Number (%)	Number (%)
**Discussed with father**			
Mean ± SD = 3.7 ± 4.5; Median = 2; Min = 0; Max = 20			
No	108 (70.1)	203 (88.3)	311 (80.9)
Yes	46 (29.9)	27 (11.7)	73 (19.1)
**Discussed with mother**			
Mean ± SD = 5.6 ± 5.0; Median = 5; Min = 0; Max = 22			
No	98 (63.6)	163 (70.9)	261 (67.9)
Yes	56 (36.4)	67 (29.1)	123 (32.1)
**Discussed with parent**			
Mean ± SD = 9.4 ± 8.8; Median = 7; Min = 0; Max = 42			
No	109 (70.8)	193 (83.9)	302 (78.6)
Yes	45 (29.2)	37 (16.1)	82 (21.3)

Outside of the family, adolescents wanted to discuss with health staff on SRH issues comprised about 59.4%, followed by with peers (47.9%) and with their teachers (32.8%). The main reason that adolescents wanted to discuss SRH issues with health staff was that health staff had more knowledge of sexual and reproductive health than them, but the highest percentage of the reason for why adolescents chose someone for discussion was that they were always a good listener for them in SRH issues.

### Attitudes of students towards general and SRH communication with parents

About 70.8% of both male and female students had positive attitudes towards and accepted the importance of maintaining discussion about general matters with their parents and more than half of adolescents agreed that their parents were good listeners. Moreover, 94.1% of students had positive attitudes towards and accepted the importance of discussing SRH issues with their parents, meaning that almost all adolescents were concerned about SRH problems and wanted to consult their parents. However, the frequency of SRH communication between parents-adolescents was low (Table 3).

**Table 3.** Attitudes towards general and SRH communication with parents.

Variable	Male (n = 154)		Female (n = 230)		Total (n = 384)	
	number	percentage	number	percentage	number	percentage
**Attitudes towards general communication with parents**						
Negative attitudes	45	29.2	67	29.2	112	29.2
Positive attitudes	109	70.8	163	70.8	272	70.8
**Attitudes towards SRH communication with parents**						
Negative attitudes	8	5.2	15	6.5	23	5.9
Positive attitudes	146	94.8	215	93.5	361	94.1

### SRH information sources

The main sources of SRH information for adolescents were health facilities because they received good information from them. In addition, the internet was cited as the second most important source of SRH information due to the fact that the internet was easy to access for SRH information (Table 4).

**Table 4.** Reasons for accessibility to SRH information sources.

Reasons for accessing source	School	Health facility	Youth center	Internet	Television	Radio	Newspaper	Magazine
	N (%)	N (%)	N (%)	N (%)	N (%)	N (%)	N (%)	N (%)
Easy to access information	73 (39.9)	91 (33.4)	52 (31.9)	98 (41.9)	57 (39.6)	31 (34.1)	42 (38.1)	39 (36.8)
Always good information	68 (37.2)	133 (48.9)	74 (45.4)	84 (35.9)	60 (41.7)	46 (50.5)	54 (49.1)	54 (50.9)
Comprehensive information	13 (7.1)	27 (9.9)	22 (13.5)	31 (13.2)	16 (11.1)	11 (12.1)	11 (10.0)	10 (9.5)
Easy to understand information	17 (9.3)	17 (6.3)	13 (7.9)	15 (6.4)	7 (4.8)	3 (3.3)	3 (2.8)	3 (2.8)
Free information	12 (6.6)	4 (1.5)	2 (1.3)	6 (2.6)	4 (2.8)	0 (0.0)	0 (0.0)	0 (0.0)
**Total**	183 (47.7)	272 (70.8)	163 (42.4)	234 (60.9)	144 (37.5)	91 (23.7)	110 (28.6)	106 (27.6)

N = number, (%) = percentage.

### Factors associated with SRH communication between students and their parents

The multivariate logistic regression model showed that being a male adolescent (AOR = 2.1; 95% CI 1.2 to 3.5), urban location (AOR = 0.2; 95% CI 0.1 to 0.5), having a mature father (AOR = 1.7; 95% CI 1.0 to 2.9), positive attitudes towards general communication with parents (AOR = 2.2; 95% CI 1.1 to 4.2) and accessibility to many sources of SRH information (AOR = 5.2; 95% CI 2.4 to 11.4) were significantly associated with adolescent-parent communication on SRH issues (p < 0.05) (Table 5).

**Table 5.** Bivariate and multivariate analysis of factors associated with SRH communication between students and their parents in Vientiane Capital.

Variable	SRH communication with parent		COR (95% CI)	AOR (95% CI)
	No (n = 302)	Yes (n = 82)		
	Number (%)	Number (%)		
**Age**				
14–15 years old	126 (79.7)	32 (20.3)	1	1
16–17 years old	176 (77.8)	50 (22.2)	1.1 (0.6–1.9)	1 (0.3–2.5)
**Sex**				
Female	193 (83.9)	37 (16.1)	1	1
Male	109 (70.7)	45 (29.3)	2.1 (1.2–3.3)*	2.1 (1.2–3.5)**
**School location**				
Rural	104 (73.2)	38 (26.8)	1	1
Urban	198 (81.8)	44 (18.2)	0.6 (0.3–1.0) *	0.2 (0.1–0.5)**
**Marital status of parents**				
Together	262 (80.1)	65 (19.9)	1	1
Separated/Divorced	40 (70.1)	17 929.9)	1.7 (0.8–3.3)	1 (0.2–4.9)
**Living arrangement**				
Both parents	254 (80.3)	62 (19.7)	1	1
Mother only or father only	35 (74.4)	12 (25.6)	1.4 (0.6–2.8)	1.4 (0.2–7.2)
Relative or friend	13 (61.9)	8 (38.1)	2.5 (1.0 − 6.3)	5.8 (1.4–22.3)
**Age of father**				
47 years or below	172 (83.5)	34 (16.5)	1	1
Above 47 years old	130 (73.1)	48 (26.9)	1.8 (1.2–3.1)*	1.7 (1.0–2.9)*
**Father’s education**				
Primary	64 (73.5)	23 (26.5)	1	1
High school/Vocational	114 (80.8)	27 (19.2)	0.6 (0.3–1.2)	1.4 (0.5–3.7)
Tertiary	139 (75.5)	32 (20.5)	0.7 (0.3–1.3)	2.7 (0.7–9.2)
**Father’s Occupation**				
Unemployed	13 (72.2)	5 (27.7)	1	1
Employee (private/govt)	150 (82.4)	32 (17.6)	0.5 (0.1–1.6)	0.5 (0.2–2.5)
Other jobs	139 (75.5)	45 (24.5)	0.8 (0.1–1.0)	0.9 (0.2–3.6)
**Age of mother**				
42 years or below	140 (79.5)	36 (20.5)	1	1
Above 42 years old	162 (77.8)	46 (22.2)	1.1 (0.6–1.8)	0.7 (0.3–1.4)
**Mother’s education**				
Primary	97 (72.9)	36 (27.1)	1	1
High school/Vocational	130 (80.7)	31 (19.3)	0.6 (0.3–1.1)	1 (0.3–2.7)
Tertiary	75 (83.3)	15 (16.7)	0.5 (0.2–1.0)	0.7 (0.3–1.5)
**Mother’s Occupation**				
Housewife	79 (75.9)	25 (24.1)	1	1
Employee (private/govt)	88 (85.4)	15 (14.6)	0.5 (0.2–1.0)	0.8 (0.3–2.3)
Other jobs	135 (76.3)	42 (23.7)	0.9 (0.5–1.7)	0.7 (0.3 − 1.5)
**Attitudes towards general communication with parent**				
Negative attitudes	97 (86.6)	15 (13.3)	1	1
Positive attitudes	205 (75.4)	67 (24.6)	2.1 (1.1–4.1)*	2.2 (1.1–4.1)*
**Accessibility to SRH information source**				
One source	114 (93.4)	8 (6.6)	1	1
>1 source	188 (71.8)	74 (28.2)	5.6 (2.5–13.9)**	5.2 (2.4–11.4)**

*significant association (p < 0.05) and **significant association (p < 0.01).

## Discussion

Parents are important role models in adolescents’ lives. They can directly or indirectly transmit values, traditions and lifestyles to their children. Positive family communication helps adolescents develop the values, security, and sense of worth that can lead to healthy decision-making including around sexual health [28]. Issues related to SRH can be difficult topics to discuss within the family. The purpose of this study was to assess the high school adolescents’ communication with their parents about sex and reproductive health, the frequency of this communication and the responses to SRH topics from the adolescents’ perspective as expressed in the questionnaire.

In this study, less than 1 in 4 adolescents discussed SRH issues regularly with their parents, which indicates that SRH issues may be difficult for parents and adolescents to discuss. The reasons for this in this study are not clear but are likely to include embarrassment, lack of confidence or socio-cultural norms that make sexual activity a taboo topic [22,29]. This is of concern as adolescents are more likely to make informed, less risky decisions about their sexual health when they have access to appropriate and timely advice, with parents being an influential source of information for their adolescent children [30–32].

This study indicated that both male and female adolescents were more comfortable discussing SRH issues with mothers than with fathers. This might be mothers were also perceived to be better at listening than fathers. Fathers may find it harder to discuss potentially embarrassing relational topics and have an open conversation about sexual health with their adolescent children and may need further support in initiating such conversations [33].

Unlike a study in southern Ethiopia [14], the present study showed that male adolescents were more likely than female adolescents to have discussed SRH issues with their parents. This might be because boys feel less embarrassment than girls when discussing SRH. It may also be because male adolescents stay at home less and go to bars and nightclubs, meaning parents may wish to talk more to their sons about contraceptive use, to prevent unwanted pregnancies [34]. Additionally, parents may not like to think of their adolescent daughters having sex, and thus may feel embarrassed to talk about sex or may fear that talking about safe sex will promote promiscuous behaviour.

Adolescents with older fathers were more likely to discuss SRH issues as found in a previous study in Unguja-Zanzibar, Tanzania [27]. This may be due to the fact that older parents were more experienced in communicating and were more open to talking with their adolescent children than younger parents.

Adolescents who have positive attitudes about communication with their parents were more likely to discuss SRH issues than those who had negative attitudes towards such communication. More than half the respondents identified that it was important to discuss SRH issues with their parents, particularly if they are good listeners. Thus, developing good listening skills is likely to motivate sons and daughters to talk with their parents [33,35], and feel more comfortable and confident to talk to their parents about SRH issues. In southern Ethiopia, students who perceived the importance of discussing SRH issues with their parents were more likely to do so compared to those who did not perceive the importance of such discussion [14].

The study also showed that adolescents who received information about SRH from multiple sources were more likely to discuss these issues with their parents. Similarly, a study in northwest Ethiopia mentioned that students who had obtained SRH information were more likely to communicate about SRH issues with their parents than those who did not have SRH information. This could be explained by the fact that the respondents have some awareness and might be more eager to communicate on SRH issues and the information they received might prepare them to begin that communication [19,27]. Adolescents attended schools in an urban area where we're less likely to communicate with parents on SRH issues than those in the rural area. One reason might be in rural settings where early marriage and childbearing are more common, parents have more liberal attitudes.

As with all studies, this study has limitations. Firstly, the sample selected only one rural and one urban school so it may not represent Lao PDR population overall. Secondly, adolescents in this study were limited to middle adolescents, aged 14 to 17. The other issue was that the responses from adolescents might be different from what their parents might really have perceived. Thirdly, our measure of sexual communication focused on several sexual risk and protective behaviours but did not assess many other topics that theorists and scholars have described as being part of sexual health such as intimacy and sexual pleasure. In addition, recall bias might have occurred as the SRH communication might or might not have happened: remembering their experiences of SRH communication with their parents during the previous six months may be inaccurate, so the adolescents’ responses might be over reported or under reported. Fourthly, the study did not capture the parents’ perspectives; however, anecdotal evidence suggested that adolescents’ perspectives are valid in parent-adolescent sexual communication.

## Conclusion

Parent-adolescent sexual and reproductive health communication was very limited and associated with sex of adolescent students, school location, attitudes of adolescents, younger age of fathers and information sources. Our findings emphasised the need to improve SRH communication for adolescents and parents in Lao PDR. Therefore, sex education should improve SRH communication skills in all schools, especially in student populations that have a high percentage of younger fathers. In addition, the programmes should involve multiple sectors like health, education and youth services to provide many sources of information to change the negative attitudes of students towards SRH communication with parents to positive ones. Moreover, parents should focus on both male and female students equally in discussing SRH issues. In particular, parents should discuss SRH issues with girls because they will bear the burden of unplanned pregnancies. Further studies should be carried out from the parents’ perspective to identify factors that affect the discussion of SRH issues between students and parents in other provinces of Laos. Qualitative research should be more in-depth in relation to the SRH topics discussed between parents and adolescents and the barriers to SRH communication between parents and adolescents.

## Paper context

Adolescent–parent sexual communication is the principal means for parents to transmit sexual values and knowledge to their children; however, the parent–adolescent SRH communication is limited. So, this study explored the gap between adolescents and their parent communication on SRH problems and factors associated with parent-adolescents sexual communication which could be the evidence-based to develop the cultural-context appropriate intervention to improve the parent-adolescent sexual communication in order to promote healthy sexual practices and to reduce risky sexual behaviour among adolescents.
